# Provings and Clinical Observations with High Potencies

**Published:** 1893-11

**Authors:** M. Macfarlan

**Affiliations:** Phila., Pa.


### PROVINGS AND CLINICAL OBSERVATIONS WITH HIGH POTENCIES.

M. Macfarlan, M. D., Phila., Pa.

### Cham.$^{94M}$.

Developed symptoms of extreme fretfulness and crying—an observation made often by other provers.

### Coffea$^{80M}$.

Cured mild hysteria in a young girl. Allays great restlessness of body with mental excitability.

### Dolichos$^{2C}$.

Cured permanently a violent case of cramps within the abdomen of a girl, who had curvature of spine; attacks two or three times daily; the trouble had existed four or five years.

### Hyosc.$^{CM}$.

Cured permanently a child, who would sob and cry a great deal at night, in her sleep, without waking.

### Iodine$^{CM}$.

Wonderfully curative in marasmus, wasting of children with aphthæ in mouth. Usually prescribed remedies had done no good.

### Kreosote$^{CM}$.

A highly curative remedy in cholera infantum of children in very hot weather. *Verified often.*

### Lycopodium$^{45CM}$.

Highly curative in sniffles of children—disposition to cold in head. The great remedy for rattling sensation in the chest; bronchitis. In pneumonia and acute bronchitis of children, and in old persons. A very reliable remehy.

Child with catarrh of chest; medicine given one-quarter hour, for six hours, she commenced to rub her nose so much and so violently, and would not stop, that the parents became alarmed at its queer conduct.

Cured a thick small circular scab on side of the nose, the old woman had it three years.

Verified often its curative power in sniffles in children.

### SULPHUR$^{CM}$.

Vomiting with partial unconsciousness and dry heat of body. Highly curative remedy in a supposed hydrocephalic condition. The child lies stupid, dull.

### SULPHURIC-ACID$^{5C}$.

Acted like magic in curing a stubborn case of bronchitis in a young child; the other symptoms of the remedy present.

### LARYNX.

### ARSENIC$^{6M}$.

Oppressed and irregular breathing.

### AMMON-CARB.$^{5C}$.

Dry teasing cough at first; loose cough after two days.

### ARNICA$^{6C}$.

Highly curative in hard, dry, tight cough.

### ARUM$^{16M}$.

For one day and a half during the proving a distressing hacking cough. This gradually improved after stopping the medicine.

Hoarseness usually quickly cured.

### BAPTIS.$^{6M}$.

The *box of the larynx is very sensitive to touch, sore to swallow or speak;* disposition to clear the throat or swallow saliva.

### BURSA.$^{2M}$.

Hoarseness mostly in the morning.

### CAUST.$^{30M}$.

Symptoms of a bad cold, severe cough, constant discharge from the nose. The cough was so severe as to rack and cause soreness in chest and abdomen.

### CIMICIFUGA-RAC.$^{95M}$.

Coughs mostly at night; cough only slightly troublesome.

### CONIUM$^{3M}$.

Troublesome teasing little cough, preventing sleep; annoying tickling sensation in the glottis.

### CINA$^{12C}$.

Very curative in the spasmodic coughs of children, occurring mostly when lying down and at night.

### DIGITALIS$^{CM}$.

Troublesome choking sensation with cough; mostly at night, and on physical exertion.

### EUPAT-PERF.$^{CM}$.

Hoarse, loses his voice often.
Coughs a good deal, short and dry efforts.
*Hoarseness*, greatest in morning when he arises, like *Caust.*

### FLUORIC-ACID$^{45M}$.

Hacking cough.

### GINSENG$^{46M}$.

Hiccoughs trouble him much during the proving; never affected so before.

### GELSEM.$^{CM}$.

Coughs much during the night.

### GANJACUM$^{5C}$.

Slight dry cough.

### HEPAR-SULPH.$^{CM}$.

Did little or no good in croup where Spong. helped quickly.

### Ipecac.$^{25M}$.

Coughs violently, aversion to food.

### Iodide of potassium$^{CM}$.

Surprising increase in weight after taking the remedy for several weeks; frequent choking sensation in his larynx; loss of voice to a slight extent.

Larynx very sore; gums and mouth sensitive; eating makes mucous surface of mouth sensitive.

### Lach.$^{CM}$.

Severe gasping at the larynx; a feeling of suffocation; thought she was dying. Cured complete loss of voice, existing for many days.

The laryngeal symptoms of Lach. are the first to be developed and most marked.

### Lactuca-viros$^{5C}$.

Talking produces a vocal sensation in chest.

### Laurocerasus$^{CM}$.

Highly curative in loose cough; expectorations profuse and dotted with small points of blood.

Its action is very marked in the later stages of pulmonary consumption.

### Mephitis$^{1M}$.

Cured a very bad hoarse, hollow cough.

### Nux-vom.$^{94M}$.

Tickling, teasing cough, of a slight kind, but persistent.

### Phosph.$^{CM}$.

Exhausted, hollow cough, with great soreness in larynx.

### Plantago-min.$^{11C}$.

She is at times so overcome that she thinks she would choke and die—the language of the prover. She thinks she cannot get enough air.

### PETROL.$^{CM}$.

Cured bad, loose, chronic coughs in many cases—generally helps consumptives for a while.

### PHYTOLLACCA$^{47M}$.

Tight dry cough with pain through her chest from middle of sternum. Whenever she lays her head down cough commences. Pains her greatly. Coughs much at night. Nausea. Muscular pains affecting the extremities attend these symptoms.

### RUMEX$^{45M}$.

Caused a constant dry cough to be loose, and although chronic, almost cured it in a week. Generally helps the cough of consumptives. Short dry hack is the usual indication.

### SULPH-ACID.

Tight dry cough. Not a prominent symptom.

### SPONGIA$^{CM}$.

After making comparative tests of Ipecac—Tartar-emetic, Iodine, Hepar, Sulphur—through a course of years, I have found Spongia to be the best remedy in croup; Aconite should be given often in the first stages.

### TARAXACUM$^{67M}$.

Coughing aggravated by eating.

### TRITICUM$^{5C}$.

Constant coughing; hoarse; raises tough phlegm; sneezing; prominent symptoms.

### TRIFOLIUM-PRETENS$^{10M}$.

In the morning when she awoke had not a particle of voice; this symptom always comes on at night; voice gone; not hoarse; has a desire to clear the throat; seems to have lost the physical power to exert herself to be heard.

### TOBACCO$^{7M}$.

Highly curative in that symptom often met with—scraping, noisy clearing of the throat, grunting before beginning a sentence as if to drive away mucus.

### UVA-URSA$^{10M}$.

Symptoms of a severe cold; throat tickles; feels like coughing constantly. COUGH a prominent symptom.

### VERBASCUM$^{45M}$.

Troublesome cough.

### ZINC-SULPH$^{5C}$.

Spasmodic coughs, worst at night.

### CHEST.

### ALETRIS$^{45M}$.

Raises much mucus; she desires to cough, but is afraid because it pains.

### ARSENIC$^{6M}$.

Great difficulty in breathing, like asthma.

### AGARIC$^{2M}$.

Soreness within the chest on either side of the sternum; hurts or weakens her to speak or breathe deeply.

### APOCYNUM$^{6M}$.

Pulse very slow.

### ARGENT-NIT.

Produced severe symptoms of angina pectoris; great difficulty of breathing, choking, etc.; pains about the heart. Very prominent symptoms.

### ARSENIC$^{6M}$.

Sensation of pressure in upper part of both lungs. Feels as if she might smother from this cause.

### Arum.$^{16M}$.

Soreness within the chest.

Pain under left short ribs.

### Borax$^{3M}$.

Soreness all over the muscles of his chest; worse about the middle of the sternum.

### Bursa.$^{9}$.

Sighing respiration.

### Antimony$^{6M}$.

Did no good in severe cases of infantile catarrh. Lyc. helped the cases immediately; true also of adults where Antimony had been used. Cepa has often helped children in such cases.

### Cornus-florid.$^{45M}$.

Sighs very often.

### Cuprum-acet.$^{45M}$.

Feels as if there was a *load at the apex of both lungs.*

### Dolichos$^{2C}$.

Sharp pain through the upper portion of both lungs.

### Doryphora$^{45M}$.

Soreness and oppression of the chest with sore throat.

### Eupat.$^{CM}$.

Pain through the right nipple when he takes a deep breath.

Oppression at the middle of the sternum; feels as if something was pressing against his heart. Hurts her to take a full breath. Chest oppressed

Oppression of chest very great.

### Eupion$^{45M}$.

Pains under both short ribs.

### FORMICA$^{45M}$.

Felt as if she would smother; cough very severe; violent straining sensation in coughing, as if the mucus would choke her; free expectoration.

### FERRUM-MET.$^{80M}$.

Stopped a daily spitting of blood and disposition to hemorrhage in a consumptive.

### GINSENG$^{46M}$.

Sneezing, cold in chest, sharp pain in his lungs, caused hoarseness with coughing.

### GETTYSBURG SPRING WATER$^{45M}$.

Considerable soreness of muscles in upper left chest; great soreness on breathing.

### HELLEBORE$^{CM}$.

Muscular pain under left nipple, some general muscular soreness over the whole body.

### IRIS$^{CM}$.

Severe *cramps* or spasms from middle of sternum to pit of stomach with repeated *vomitings*.

### KALI-CARB.$^{24M}$.

Shooting pains within his chest like pleurisy.

### KAOLIN$^{45M}$.

Muscular soreness at apex of both lungs, with some lung pain.

Remarkable in relieving great soreness of lungs from cold; breathing very painful.

The symptoms had lasted some days and relief was rapid.

### KALI-HYD.$^{CM}$.

Had to rise from bed, thinking he would be smothered.

Pains through both lungs, feels tired and weak, fluttering sensation at heart and very nervous.

Sharp pains through the right lung from the nipple backward; general distressed feeling within the chest.

LACTUCA-VIROS.$^{5C}$.

Sudden sharp pains affect his lungs, walls of his chest front and back sore.

Soreness in lungs, which seem sensitive, on deep breathing.

LILIUM-TIG.$^{45M}$.

Smothering sensation in chest. No pain; a spasmodic or nervous sensation.

LACH.$^{CM}$.

Caused suffocative sensation, distress referable mostly on either side of middle of sternum.

LYCOPODIUM$^{45M}$.

Given every two hours for a week; caused great pain in the left side of the chest; could breathe only with the greatest pain and difficulty.

A most reliable and curative remedy in pneumonia, of children particularly.

Wonderful in relieving the distress with shortness of breath of pneumonia, when *Nitrum*, Phosphorus, and other remedies did no good.

NITRUM$^{5C}$.

Arrested quickly the most severe attack of asthma. This observation, made twenty years ago, has been verified scores of times.

NUX-VOM.$\theta$.

Produced great sneezing; gaping frequently, as if sleepy.

PLANTAGO-MIN.$^{11C}$.

Short-breathed, spasmodic symptom.

Choking sensation as if from grief.

PULSATILLA$^{CM}$.

Pains within the chest at both nipples; slight cough; giddiness; loss of appetite.

PHOSPH.$^{CM}$.

Pains down middle of sternum to epigastrium.

PSORIN.$^{42M}$.

Soreness to the touch below the middle of the sternum; troubled greatly with wind; breathing oppressed as if from wind.

PIX-LIQUIDA$^{5C}$.

Raised stringy mucus with bright-red blood; cough constant.

MERC-VIV.$^{101M}$.

Soreness within from the larynx to the ensiform cartilage; felt mostly on swallowing, which is very painful. No soreness on deep breathing.

SERPENTARIA$^{5C}$.

Symptoms of cold in chest with cough.

SAPO-SODA$^{2C}$.

Sensation of cold in chest, which feels sore and bruised; pains her to cough; coughs a good deal; feeling of tightness in middle of sternum.

SARRACENIA$^{2M}$.

Sharp pain right through the right lung to his back, commencing three inches below right nipple, and running directly backward to the scapala.

